# Soil Microbiomes From Fallow Fields Have Species-Specific Effects on Crop Growth and Pest Resistance

**DOI:** 10.3389/fpls.2020.01171

**Published:** 2020-08-05

**Authors:** Mia M. Howard, Christian A. Muñoz, Jenny Kao-Kniffin, André Kessler

**Affiliations:** ^1^ Plant Biology Section, School of Integrative Plant Science, Cornell University, Ithaca, NY, United States; ^2^ College of Human Ecology, Cornell University, Ithaca, NY, United States; ^3^ Horticulture Section, School of Integrative Plant Science, Cornell University, Ithaca, NY, United States; ^4^ Department of Ecology and Evolutionary Biology, Cornell University, Ithaca, NY, United States

**Keywords:** aboveground-belowground interactions, agricultural microbiome, herbivore resistance, old-field succession, plant-soil feedbacks, rhizosphere, *Spodoptera frugiperda*, *Trichoplusia ni*

## Abstract

Communities of microorganisms in the soil can affect plants’ growth and interactions with aboveground herbivores. Thus, there is growing interest in utilizing soil microbiomes to improve plant performance in agriculture (*e.g.*, for pest control), but little is known about the phenotypic responses of various crop species to different microbiomes. In this study, we inoculated four crop species from different botanical families, maize (*Zea mays*, Poaceae), cucumber (*Cucumis sativus*, Cucurbitaceae), tomato (*Solanum lycopersicum*, Solanaceae), and lettuce (*Lactuca sativa*, Asteraceae), with diverse soil microbiomes originating from actively-managed agricultural fields or fallow fields under varying stages of succession (1, 3, and 16-years post-agriculture) sourced from a large-scale field experiment. We compared the crops’ responses to these different microbiomes by assessing their growth and resistance to two generalist insect pests, cabbage looper (*Trichoplusia ni*) and fall armyworm (*Spodoptera frugiperda*). These different microbiomes affected both plant growth and resistance, but the effects were species-specific. For instance, lettuce produced the largest leaves when inoculated with a 3-year fallow microbiome, the microbiome in which cucumber performed worst. Plants were generally more resistant to *T. ni* when inoculated with the later succession microbiomes, particularly in contrast to those treated with agricultural microbiomes. However, for tomato plants, the opposite pattern was observed with regard to *S. frugiperda* resistance. Collectively, these results indicate that plant responses to microbiomes are species-specific and emphasize the need to characterize the responses of taxonomically diverse plant species to different microbiomes.

## Introduction

Some microorganisms in the soil can improve the performance of plants, and hence, there is growing interest in manipulating soil microbiomes to improve yield and pest control in agriculture (Bell et al., 2019). Soil microbiomes can promote plant growth by enhancing host tolerance to abiotic stresses, such as drought (Lau and Lennon, 2012), as well as to biotic stresses, such as pathogens and herbivores. Microorganisms can affect plants’ resistance to pathogens and herbivores through altering secondary metabolite production, as well as inducing plant defense responses (Ludwig-Müller, 2015; Harun-Or-Rashid and Chung, 2017). One recent study suggests that the rhizosphere microbiome may even serve as a stronger driver than plant genetics in determining plant resistance to insect herbivores (Hubbard et al., 2019). Thus, the soil microbiome is frequently proposed as a target for improving pest management in agriculture (Pineda et al., 2017).

Agricultural cultivation alters soil conditions, which can have long-term negative consequences for plant performance. These disturbances can be caused by multiple conventional practices such as tillage and fertilizer application (reviewed in Howard et al., 2019) and greater agricultural intensification (*i.e.*, conventional versus organic farming) has been associated with lower levels of beneficial soil microbes, such as arbuscular mycorrhizal fungi, and decreased complexity of fungal networks (Banerjee et al., 2019). In some cases, continuous cultivation of crop monocultures can result in the build-up of pathogens in the soil, which is often implicated as a causal agent of “replant disease”, reducing yields of a broad range of crops from annuals, such as maize, to tree fruits (Traquair, 1984; Bennett et al., 2012). This condition is typically avoided by reducing the abundance of suitable plant hosts through crop rotation or polyculture (Bennett et al., 2012). With regard to insect pests, one recent study suggests that the lower insect resistance of plants in conventionally versus organically managed fields is at least partially due to differences in soil microbial communities (Blundell et al., 2020). Recent work has also shown that soil microbiomes conditioned by non-crop plants, such as grassland species, can reduce the susceptibility of chrysanthemums to both pathogens and insect herbivores (Ma et al., 2017; Ma et al., 2019; Pineda et al., 2020), suggesting that soil microbiomes from natural systems could be used to improve crop performance.

Leaving fields fallow and promoting the establishment of biota unimpeded by tillage and other disruptive management practices not only results in drastic changes in plant communities, but also in soil quality and soil microbiomes. Levels of soil organic matter, nutrients, and microbial biomass increase in cultivated fields that are left fallow for extended lengths of time (Post and Kwon, 2000; Howard et al., 2020) and these successional changes in soils may affect plant growth and resistance to herbivores (Howard et al., 2018). The composition of soil microbiomes is also widely known to shift over ecological succession (Maharning et al., 2009), which is likely to affect the performance of the plants with which they interact. For example, the abundance of pathogenic fungi has been found to decrease over successional time in abandoned agricultural fields (Hannula et al., 2017), suggesting that these shifts are functional and may benefit plants over succession. Our recent work also suggests that these successional shifts in microbial communities may improve plants’ resistance to herbivores (Howard et al., 2020).

We previously found that the rhizosphere microbial communities of a native plant, tall goldenrod, *Solidago altissima* (Asteraceae), shift over oldfield succession in fallow maize fields, with functional effects on their resistance to herbivores. When we inoculated *S. altissima* with soil microbiomes from a plant community that had been left fallow for 15 years, these plants were more resistant to the specialist goldenrod leaf beetle, *Trirhabda virgata*, than their counterparts inoculated with early succession microbiomes, paralleling the pattern of greater herbivore resistance observed among goldenrods in late succession communities (Howard et al., 2020). However, it is not known whether this microbiome-mediated resistance associated with the later oldfield succession soils is specific to the community-dominating goldenrods or could more broadly enhance the insect resistance of other plant species as well.

Little is known about the consistency of phenotypic responses of diverse plant species to whole soil microbial communities in terms of plant performance and especially herbivore resistance. The broad effectiveness of individual growth-promoting microbes across plant species has been demonstrated for several “beneficial” microbes, such as mycorrhizae and other fungal endophytes, which can promote the growth of annual crops and trees alike, albeit to different degrees (Munyanziza et al., 1997; Khan et al., 2012; Van Geel et al., 2016). Yet, while diverse plant species assemble different microbiomes—likely influenced by their phylogenetic relatedness (Fitzpatrick et al., 2018), interspecific comparisons of phenotypic responses to whole soil microbiomes are limited and have shown that responses can vary by plant species (Fitzpatrick et al., 2018; Hahl et al., 2019). However, Fitzpatrick et al. (2018) found that plants grew larger in soil microbial communities conditioned by plant species with microbiomes that were more dissimilar to their own, suggesting that plant species responses may be predictable.

Understanding the predictability of plant responses to microbiomes will be important for assessing the potential and general applicability of microbiome-manipulations in agriculture, including the applications of findings based on native plants and non-crop models to agronomically important species. Panke-Buisse et al. (2015) found that complex soil microbiomes that were artificially selected to promote earlier flowering time in *Arabidopsis thaliana* effectively decreased the latency of flowering in the confamilial crop *Brassica rapa*. Yet, whether these same microbiomes would similarly manipulate the phenology of more distantly related species is unknown. In addition to variation driven by phylogenetic distance, there may be differences in plant-microbe interactions in crop plants versus wild plants due to domestication, particularly breeding under high-input conventional agricultural conditions in which forming symbioses with mutualist microorganisms may not be as crucial for plants as in natural systems (Pérez-Jaramillo et al., 2016; Porter and Sachs, 2020).

In this study, we sought to examine whether the microbiome-mediated trend in herbivore resistance that we discovered in an ecologically important native plant, *S. altissima*, could be applied to manipulate the pest resistance of crop species. We selected four crop species from different families: maize (*Zea mays*, Poaceae), cucumber (*Cucumis sativus*, Cucurbitaceae), tomato (*Solanum lycopersicum*, Solanaceae), and lettuce (*Lactuca sativa*, Asteraceae) and inoculated them in a glasshouse with soil microbiomes collected from experimental field plots that were currently under cultivation (conventional maize agriculture) or had been fallow for 1, 3, or 16 years. We assessed their early-season growth and resistance to two agricultural pests, *Spodoptera frugiperda* (fall armyworm) and *Trichoplusia ni* (cabbage looper). Based on our finding that late succession soil microbiomes conferred the greatest herbivore resistance to *S. altissima* (Howard et al., 2020), we predicted that the crop plants would be most resistant to the insect pests when inoculated with the 16-year fallow microbiome.

## Materials and Methods

### Plant Materials

We obtained crop seeds (one cultivar per species) from Burpee Co. (Warminster, PA, USA): Sweet Sunshine Hybrid sweet corn (maize), organic Roma tomato, Pick-a-Bushel Hybrid cucumber, and Parris Island Cos lettuce.

### Successional Inoculants

We obtained soil microbiome inoculants from fields that had undergone three consecutive years of conventional maize cultivation (year “0”) and plant communities in the 1^st^, 3^rd^, and 16^th^ years of fallow (oldfield) succession from a large-scale successional field experiment at Dunlop Meadow in Brooktondale, NY, USA (42°23’13”N, 76°24’00”W) (described in Howard et al., 2020). Briefly, this field experiment consisted of duplicated 30 x 30 m plots in which maize is grown conventionally for 3 years and then left fallow. The plantings are staggered chronologically so that plots in different years of fallow succession can be sampled simultaneously and there are two plots representing each successional year. On May 22, 2019, we collected soils for use as inoculants from the top 10 cm of each plot. To generate an inoculant that was representative of the plot, we sampled soil from 5 locations within each plot, homogenized these subsamples in a plastic bag, and sieved them to 4.75 mm. We stored these soils at 4°C for one day before using them to inoculate sterilized soil. Based on our previous surveys of these plots (Howard et al., 2020), we expect these inoculants to vary substantially in the composition of their bacterial and fungal communities.

### Plant Inoculation and Growth

We transferred the successional soil microbiomes to sterilized potting media by directly inoculating a mix of triple autoclaved (with 24 h rest periods in between cycles) commercial sphagnum moss potting media (75% (v/v)) (Lambert’s All Purpose, Quebec, Canada) and topsoil (20% (v/v)) with each field soil inoculant at a rate of 5% (v/v), an inoculation method which we previously optimized for this recipient soil type (Howard et al., 2017). As our objective was to compare plant responses to microbiomes from agricultural fields with those from communities in different stages of fallow succession, rather than the effect of inoculation more generally, we did not include a sterile or mock inoculation treatment. We prepared six replicate pots of inoculated soil per inoculant, for a total of 12 pots per successional inoculant year, watered them with filter-sterilized deionized water (0.1 μm pore size, Sawyer Products, Inc., Florida, USA) to a moisture level of approximately 10% (v/v) and allowed the pots to incubate at ambient temperature (~ 27°C) in a glasshouse at Cornell University (Ithaca, NY, USA) for 24 h prior to planting. We surface-sterilized seeds in an aqueous solution containing 1% (w/v) NaOCl (diluted from household bleach) and 0.0042% (v/v) Tween20 for 10 min prior to planting on 24-May-2019 to minimize the effect of the existing microbes colonizing them. We planted three seeds in each 10 cm diameter pot (ultimately thinned to one plant—the largest seedling—per pot) to a depth of 25 mm for maize and cucumber and 6 mm for tomato and lettuce and positioned the pots in a randomized block design in a glasshouse with a 16 h photoperiod. We irrigated the pots with filter-sterilized water as needed and removed any weeds that germinated from the seedbank of the inoculant soil.

### Plant Growth and Measurements

As measures of plant size, we recorded the number of leaves and length of the longest leaf of each maize and cucumber plant 20 days after planting (DAP), and for each tomato and lettuce plant 23 and 31 DAP, respectively. To obtain an approximate final biomass (roots and shoots, minus the tissues collected for bioassays and analysis) of each plant, we harvested the maize, cucumber, tomato, and lettuce plants 32, 32, 33, and 39 DAP, respectively, washed the soil off of their roots, and weighed them after drying in an oven at 60°C for 7 days. At the time of harvest, two of the largest leaves were already removed for use in herbivore resistance bioassays (see below). We also measured specific leaf area (SLA) at harvest by punching two 10 mm diameter leaf discs from the youngest fully expanded leaves of maize, cucumber, and tomato plants and the second fully-expanded leaves of lettuce, and dividing the area of these discs by their dry weight.

### Herbivore Resistance Bioassays

We obtained *Trichoplusia ni* (cabbage looper) and *Spodoptera frugiperda* (fall armyworm) as eggs from Benzon Research, Inc. (Carlisle, PA, USA) and reared them on cabbage looper diet (Southland Projects, Inc., Lake Village, AR, USA) prepared according to the manufacturer’s instructions.

#### Choice Bioassays

To assess the feeding preference of herbivores for plants grown with agricultural or fallow microbiomes, we conducted two-way cafeteria choice tests with *T. ni* and *S. frugiperda* (illustrated in **Figures 3A** and **5A**). In each test, we simultaneously presented a neonate larva (*c.* 2 days post-hatching) with 7 mm diameter leaf discs (punched from the youngest fully-expanded leaf, or the first collared leaf for maize) from a plant inoculated with an agricultural (year 0) and a fallow (year 1, 3, or 16) microbiome, in a 118 mL soufflé cup (Solo Cup Co., Urbana, IL, USA) with a thin layer of agar (12.5 g/L) at the bottom to prevent desiccation. We recorded the first disc the larvae were observed to eat and if they did not feed within 2 h, we excluded the test from the analysis. We randomly paired each agricultural microbiome-inoculated plant with a plant from each of the three successional year treatments and tested each of these pairings twice with individual, naïve larvae of each herbivore species (N = 20-24 tests per plant species × fallow inoculant age level × herbivore species combination). We performed the choice tests 21, 21, 23, and 33 DAP for maize, cucumber, tomato, and lettuce, respectively.

#### No-Choice Bioassays

As another measure of herbivore resistance, we performed no-choice feeding assays in which we offered *T. ni* and *S. frugiperda* larvae (*c.* 6 days post-hatching) leaf tissue from a single plant in individual agar cups (as in the choice assay). This piece of tissue was excised from the second youngest collared leaf for maize (mean ± SE: 9.76 ± 0.2 cm^2^), half of the youngest uncurled leaf for cucumber cut down the midvein (16.1 ± 0.5 cm^2^), one of the side leaflets from the second youngest fully-expanded leaf for tomato (10.6 ± 0.3 cm^2^), or half of the second youngest fully-expanded leaf for lettuce cut down the midvein (18.8 ± 0.5 cm^2^). We performed the no-choice assays 26 DAP for the maize, cucumber, and tomato plants and 35 DAP for the lettuce plants. We measured the amount of weight gained and leaf tissue eaten by the larvae after 3 d for maize, cucumber, and tomato, and after 4 d for lettuce. We quantified the area of leaf tissue eaten using Adobe Photoshop. As an integrated measure of herbivore performance, we calculated biomass accumulation efficiency by dividing the amount of weight gained by the area of leaf tissue consumed.

### Statistical Analyses

We performed all statistical analyses using R version 3.6.2 (R Core Team, 2019). We analyzed the plant size and no-choice bioassay data using linear mixed effects models using the function *lmer* in the package *lme4* (Bates et al., 2015) with fixed effects of inoculant age, plant species, their interaction, and inoculant source (spatial block in the field) and a random effect of greenhouse block (position in the greenhouse experiment). In addition to these cross-species analyses, we separately analyzed the plant size and no-choice bioassay data for each crop, using the same model design minus the plant species fixed effect (and interaction term). We omitted one maize and one tomato plant that died from the 1- and 3-year microbiome treatment groups, respectively. We also removed insects that died, lost weight, or did not consume any tissue in the no-choice bioassays. If necessary, we used logarithmic or square-root transformations to meet the assumptions of normality and homoscedasticity of residuals. We assessed the significance of the fixed effects using F-tests with Kenward-Rogers approximated degrees of freedom *via* the *anova* function in the package *lmerTest* (Kuznetsova et al., 2017) and used the *emmeans* function in the package *emmeans* (Lenth, 2020) to examine pairwise contrasts between the different inoculant age levels. We analyzed the choice bioassay data using generalized linear mixed-effects models (family = binomial) with choice as a binary variable (agricultural microbiome vs. fallow microbiome), inoculant successional age, crop species, and their interaction as fixed effects, and individual plants (both agricultural and fallow microbiome-inoculated) as random effects using *glmer* in the package *lme4*. Additionally, we separately analyzed choice data for each crop species, using the same model design minus the crop species fixed effect (and the interaction term). We calculated 95% confidence intervals for the probabilities of eating a disc from each inoculant treatment level using *emmeans* and then determined whether or not insects showed a significant preference based on whether this interval included 0.5 (where the probabilities of choosing the agricultural microbiome-inoculated plant vs. the fallow microbiome-inoculated plant are equal).

## Results

**Figure 1:**
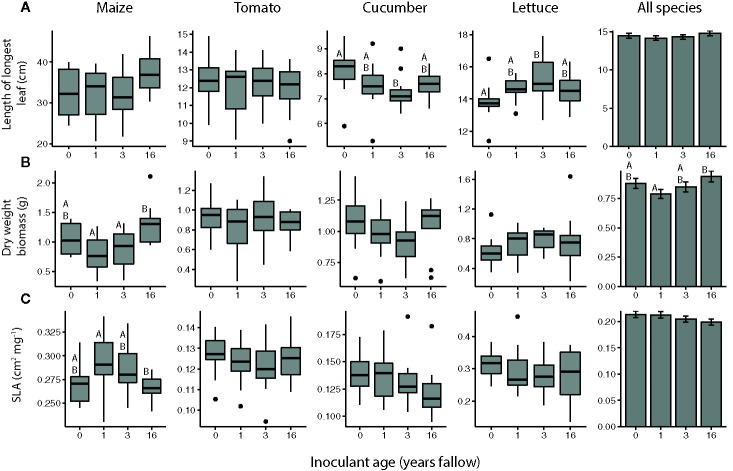
Sizes of maize (*Z. mays*), tomato (*S. lycopersicum*), cucumber (*C. sativus*), and lettuce (*L. sativa*) plants inoculated with agricultural (0 year) or fallow microbiomes of different ages (1, 3, or 16 years post-agriculture) measured as **(A)** the length of the longest leaf, **(B)** dry weight biomass (roots and shoots), and **(C)** specific leaf area (SLA). All plants (N = 11-12 per species × inoculant age treatment) were grown in a glasshouse common garden experiment. Note that 2 leaves had been harvested prior to the dry weight measurement for use in the herbivore resistance bioassays. Boxes enclose the middle 50% of values, with the 50^th^ percentile indicated by the midline and error bars spanning 1.5 times the interquartile range in both directions; values outside this range are indicated as black dots. Bar plots show estimated marginal means (± SE) averaged across species, and inoculant and greenhouse spatial blocks (see model outputs in **Table 1**). Letters above the boxes/bars indicate significant differences between inoculant age levels within plant species for the boxplots and across all species for the bar plots (α = 0.05).

Plant growth was a function of inoculant successional age treatment for some crops, but the effects varied both in pattern and by growth measurement (**Figure 1**, **Table 1**). Plants generally produced the most biomass when inoculated with the oldest soil microbiome, but there were significant crop species by inoculant interactions for both leaf size and total plant biomass (**Table 1**). While inoculant successional age had a significant effect on the leaf sizes of both cucumber and lettuce plants, cucumber produced the largest leaves when inoculated with agricultural soil microbiomes and the smallest leaves when inoculated with soil from fields that had been fallow for 3 years, the opposite of the pattern observed for lettuce (**Figure 1A**; cucumber: inoculant age: *F_3,41_*= 2.8500, *P* = 0.0491, inoculant source: *F_1,41_*= 1.0906, *P* = 0.3025; lettuce: inoculant age: *F_3,41_*= 3.5328, *P* = 0.0229, inoculant source: *F_1,41_*= 1.7969, *P* = 0.1875). The dry weight biomass of cucumber and lettuce paralleled these leaf length trends, but did not differ significantly with regard to inoculant age for these species (**Figure 1B**). Maize biomass varied with inoculant successional age, growing largest when inoculated with the oldest, 16-year fallow microbiome (**Figure 1B**; inoculant age: *F_3,40_*= 5.5873, *P* = 0.0027, inoculant source: *F_1,40_*= 0.5276, *P* = 0.4718). Maize plants in this inoculation treatment also tended to have the largest leaves, and there was also an effect of inoculant source on leaf size (**Figure 1A**, inoculant age: *F_3,40_*= 2.4187, *P* = 0.0803, inoculant source: *F_1,40_*= 4.8069, *P* = 0.0342). Moreover, maize SLA varied with inoculant age, whereby larger, late-succession microbiome plants had the lowest SLA (**Figure 1C**, inoculant age: *F_3,40_*= 3.8493, *P* = 0.0164, inoculant source: *F_1,40_*= 0.8477, *P* = 0.3627). SLA did not differ significantly by inoculant age for any other crop species. In contrast to the other species, none of the growth measurements of tomato plants were affected by inoculant age (**Figure 1**).

**Table 1:** Results of ANOVAs assessing the overall effects of soil microbial inoculants on plant growth across crop species.

Growth measurement	Variable	*F*	*P*
Leaf length	Inoculant age	*F_3,165_* = 1.0429	0.3752
	Crop species	*F_3,8_*= 442.9242	<0.0001***
	Inoculant source	*F_1,165_* = 2.1440	0.1450
	Inoculant age × crop species	*F_9,165_* = 2.3019	0.0184*
Dry weight biomass	Inoculant age	*F_3,165_* = 2.9407	0.0348*
	Crop species	*F_3,8_*= 5.0985	0.0291*
	Inoculant source	*F_1,165_* = 1.7916	0.1826
	Inoculant age × crop species	*F_9,165_* = 2.3807	0.0147*
SLA	Inoculant age	*F_3,165_* = 1.8785	0.1352
	Crop species	*F_3,8_* = 152.0031	<0.0001***
	Inoculant source	*F_1,165_* = 0.0601	0.8067
	Inoculant age × crop species	*F_9,165_* = 1.1778	0.3123

Maize (*Z. mays*), tomato (*S. lycopersicum*), cucumber (*C. sativus*), and lettuce (*L. sativa*) plants were inoculated with soil microbiomes from agricultural (0 years) or fallow plots of different ages (1, 3, or 16 years post-agriculture), with 2 plots representing each age (inoculant source). Plant growth was measured as the length of the longest leaf, dry weight biomass (roots and shoots), and specific leaf area (SLA). N = 11-12 per species × inoculant age treatment. Note that 2 leaves had been harvested prior to the dry weight measurement for use in the herbivore resistance bioassays. *p < 0.05, ***p < 0.001.

**Figure 2:**
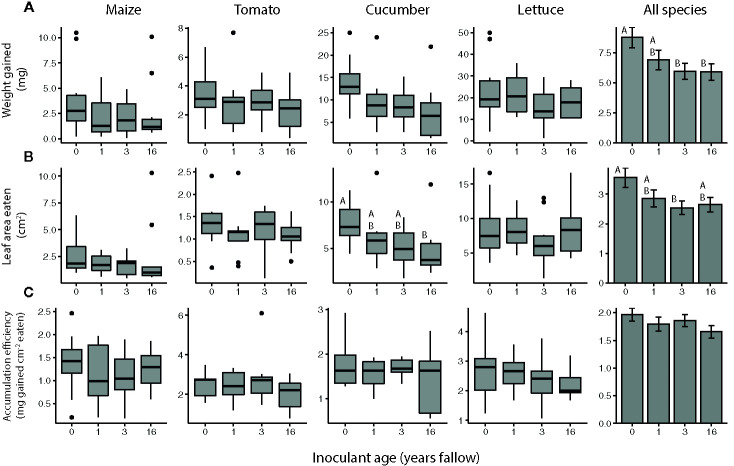
*Trichoplusia ni* resistance of maize (*Z. mays*), tomato (*S. lycopersicum*), cucumber (*C. sativus*), and lettuce (*L. sativa*) plants inoculated with agricultural (0 year) or fallow microbiomes of different ages (1, 3, or 16 years post-agriculture) in a no-choice assay. Resistance measured as **(A)** weight gained **(B)** amount of tissue eaten by *T. ni* larvae (*c.* 6* *d old at start), as well as their **(C)** biomass accumulation efficiency after feeding for 3 (maize, tomato, and cucumber assays) or 4 d (lettuce assays). N = 8-12 replicates per species × inoculant age treatment after omitting insects that died, lost weight, or did not eat. Boxes enclose the middle 50% of values, with the 50^th^ percentile indicated by the midline and error bars spanning 1.5 times the interquartile range in both directions; values outside this range are indicated as black dots. Bar plots show estimated marginal means (± SE) averaged across species, and inoculant and greenhouse spatial blocks (see model outputs in **Table 2**). Letters above the boxes/bars indicate significant differences between inoculant age levels within plant species for the boxplots and across all species for the bar plots (α = 0.05).

Overall, the resistance of plants to *T. ni* varied by both species and inoculant age based on the no-choice feeding experiments (**Figure 2**, **Table 2**). The agricultural microbiomes conferred the least resistance to the plants, with *T. ni* larvae gaining the most weight, and consuming the greatest amount, while feeding on the plants inoculated with agricultural soil in the no-choice experiments (**Figure 2**, **Table 2**). However, when looking at the crop species individually, we only observed a significant effect of inoculant age on the resistance of cucumber plants. *Trichoplusia ni* consumed less leaf tissue from cucumber plants inoculated with the late succession (16-year) soil compared to those treated with the agricultural microbiome (**Figure 2B**; inoculant age: *F_3,29_*= 3.0218, *P* = 0.0456, inoculant source: *F_1,28_*= 3.9438, *P* = 0.0568). Consistent with this consumption trend, the larvae also tended to gain less weight feeding on these later succession-inoculated cucumber plants, though there was not a statistically significant effect of inoculant age (**Figure 2A**; inoculant age: *F_3,29_*= 2.1902, *P* = 0.1105, inoculant source: *F_1,28_*= 3.0123, *P* = 0.0935) and there was no difference in biomass accumulation efficiency (**Figure 2C**, inoculant age: *F_3,29_*= 0.7550, *P* = 0.5285, inoculant source: *F_1,28_*= 0.0101, *P* = 0.9206).

**Table 2:** Results of ANOVAs assessing the overall effects of soil microbial inoculants on resistance to T. ni in a no-choice test across crop species.

Resistance measurement	Variable	*F*	*P*
Weight gain	Inoculant age	*F_3,131_* = 3.1318	0.0279*
	Crop species	*F_3,8_* = 86.9621	<0.0001***
	Inoculant source	*F_1,131_ = *2.4207	0.1222
	Inoculant age × crop species	*F_9,132_* = 0.6468	0.7552
Leaf area eaten	Inoculant age	*F_3,131_* = 2.7717	0.0441*
	Crop species	*F_3,8_* = 83.2206	<0.0001***
	Inoculant source	*F_1,130_* = 1.9539	0.1645
	Inoculant age × crop species	*F_9,131_* = 0.4388	0.9118
Accumulation efficiency	Inoculant age	*F_3,131_* = 1.3854	0.2501
	Crop species	*F_3,8_* = 23.7509	0.0003***
	Inoculant source	*F_1,130_* = 0.2690	0.6049
	Inoculant age × crop species	*F_9,131_* = 0.7149	0.6945

Maize (*Z. mays*), tomato (*S. lycopersicum*), cucumber (*C. sativus*), and lettuce (*L. sativa*) plants were inoculated with soil microbiomes from agricultural (0 years) or fallow plots of different ages (1, 3, or 16 years post-agriculture), with 2 plots representing each age (inoculant source). Herbivore resistance was measured in no-choice bioassays with *c.* 6 d old T. ni larvae, in which weight gain, leaf area eaten, and biomass accumulation efficiency were measured after feeding for 3 (maize, tomato, and cucumber assays) or 4 d (lettuce assays). N = 8-12 replicates per species × inoculant age treatment after omitting insects that died, lost weight, or did not eat. *p < 0.05, ***p < 0.001.

**Figure 3:**
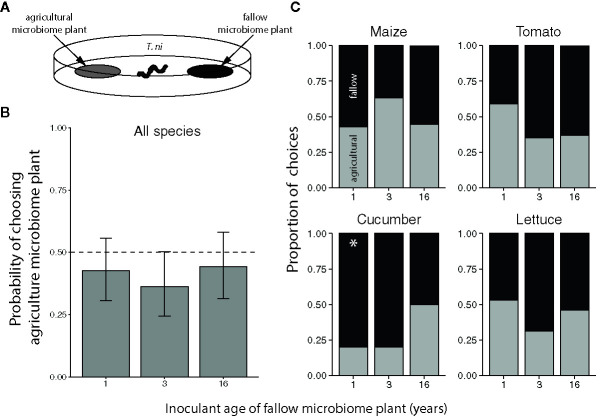
Feeding preference of *Trichoplusia ni* for maize (*Z. mays*), tomato (*S. lycopersicum*), cucumber (*C. sativus*), and lettuce (*L. sativa*) inoculated with agricultural or fallow microbiomes of different ages. **(A)** Neonatal *T. ni* larvae were simultaneously presented with a disc of leaf tissue from a plant inoculated with an agricultural soil microbiome and a disc of leaf tissue from a plant inoculated with a microbiome from a fallow field (1, 3, or 16 years post-agriculture) in an arena. All choice tests were performed within plant species. Choices represent the microbiome treatment of the disc each larva was first observed to eat. **(B)** Probabilities (with 95% confidence intervals) of selecting the agriculture microbiome-inoculated plant averaged across plant species; the dashed line indicates an equal preference for agriculture- and fallow-microbiome treated plants. **(C)** Choices by crop species; light grey bars indicate the proportion of larvae that chose the plant treated with the agricultural soil microbiome whereas the dark grey bars indicate instances in which the fallow microbiome plant was chosen. N = 9-22 tests per plant species × fallow inoculant age level after omitting larvae that did not feed within the 2 h trial. An asterisk (*) indicates a significant preference.

*Trichoplusia ni* larvae generally did not exhibit a preference for feeding on crops inoculated with agricultural versus fallow soil microbiomes, regardless of plant species or the successional age of the fallow inoculant (**Figure 3**). However, *T. ni* exhibited a marginal preference for crops with 3-year fallow microbiomes over those inoculated with agricultural soil (**Figure 3B**, probability of selecting the agricultural microbiome plant: 0.3627 with an upper 95% confidence limit of 0.5020). Yet, when looking at individual crop species, the only significant preference observed was for 1-year fallow inoculated cucumber plants; larvae selected the agricultural microbiome cucumber with a probability of only 0.2 (95% confidence interval: 0.0659 to 0.470) when tested against their 1-year fallow-inoculated counterparts (**Figure 3C**).

**Figure 4:**
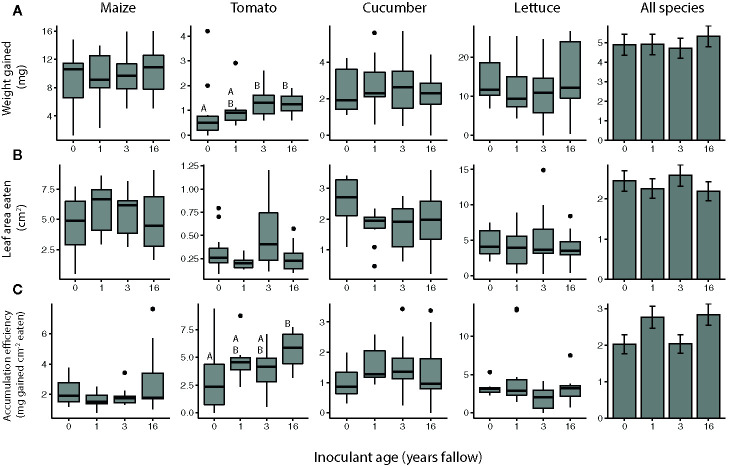
*Spodoptera frugiperda* resistance of maize (*Z. mays*), tomato (*S. lycopersicum*), cucumber (*C. sativus*), and lettuce (*L. sativa*) plants inoculated with agricultural (0 year) or fallow microbiomes of different ages (1, 3, or 16 years post-agriculture) in a no-choice assay. Resistance measured as **(A)** weight gained **(B)** amount of tissue eaten by *S. frugiperda* larvae (*c.* 6 d old at start), as well as their **(C)** biomass accumulation efficiency after feeding for 3 (maize, tomato, and cucumber assays) or 4 d (lettuce assays). N = 8-12 replicates per species × inoculant age treatment after omitting insects that died. Boxes enclose the middle 50% of values, with the 50^th^ percentile indicated by the midline and error bars spanning 1.5 times the interquartile range in both directions; values outside this range are indicated as black dots. Bar plots show estimated marginal means (± SE) averaged across species, and inoculant and greenhouse spatial blocks (see model outputs in **Table 3**). Letters above the boxes indicate significant differences between inoculant age levels within plant species (α = 0.05).

The inoculation treatments generally did not affect resistance to *S. frugiperda* in the no-choice experiments (**Figure 4**, **Table 3**), except for one species: tomato. The larvae gained more weight feeding on tomato plants inoculated with microbiomes from fields that had been fallow for 3 and 16 years compared to those treated with agricultural microbiomes (**Figure 4A**; inoculant age: *F_3,36_*= 3.8566, *P* = 0.0173, inoculant source: *F_1,36_*= 0.1336, *P* = 0.7169). While the larvae did not consume significantly greater amounts of leaf tissue from these later succession microbiome-inoculated plants (**Figure 4B**), they gained weight more efficiently feeding on the 16-year fallow microbiome plants compared to their agricultural counterparts (**Figure 4C**; inoculant age: *F_3,36_*= 4.3989, *P* = 0.0099, inoculant source: *F_1,36_*= 0.1320, *P* = 0.7185). The biomass accumulation efficiency of *S. frugiperda* varied by inoculant age, overall, though the response differed by plant species (**Table 3**, **Figure 4**).

**Table 3:** Results of ANOVAs assessing the overall effects of soil microbial inoculants on resistance to S. frugiperda in a no-choice test across crop species.

Resistance measurement	Variable	*F*	*P*
Weight gain	Inoculant age	*F_3,140_*= 0.2579	0.8556
	Crop species	*F_3,8_*= 72.5039	<0.0001***
	Inoculant source	*F_1,141_*= 0.3924	0.5320
	Inoculant age × crop species	*F_9,140_ = *0.8693	0.5543
Leaf area eaten	Inoculant age	*F_3,140_*= 0.6133	0.6075
	Crop species	*F_3,8_*= 56.0374	<0.0001***
	Inoculant source	*F_1,141_*= 0.3841	0.5364
	Inoculant age × crop species	*F_9,140_ = *0.9578	0.4777
Accumulation efficiency	Inoculant age	*F_3,140_*= 3.6408	0.0144*
	Crop species	*F_3,8_*= 8.5635	0.0071**
	Inoculant source	*F_1,140_*= 0.0109	0.9171
	Inoculant age × crop species	*F_9,140_*= 3.0900	0.0021**

Maize (*Z. mays*), tomato (*S. lycopersicum*), cucumber (*C. sativus*), and lettuce (*L. sativa*) plants were inoculated with soil microbiomes from agricultural (0 years) or fallow plots of different ages (1, 3, or 16 years post-agriculture), with 2 plots representing each age (inoculant source). Herbivore resistance was measured in no-choice bioassays with *c.* 6 d old S. frugiperda larvae, in which weight gain, leaf area eaten, and biomass accumulation efficiency were measured after feeding for 3 (maize, tomato, and cucumber assays) or 4 d (lettuce assays). N = 8-12 replicates per species × inoculant age treatment after omitting insects that died, lost weight, or did not eat. *p < 0.05, ***p < 0.001.

**Figure 5:**
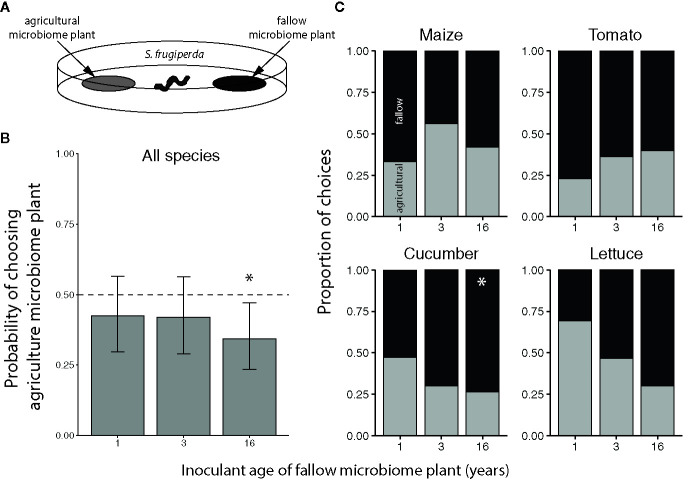
Feeding preference of *Spodoptera frugiperda* for maize (*Z. mays*), tomato (*S. lycopersicum*), cucumber (*C. sativus*), and lettuce (*L. sativa*) inoculated with agricultural or fallow microbiomes of different ages. **(A)** Neonatal *S. frugiperda* larvae were simultaneously presented with a disc of leaf tissue from a plant inoculated with an agricultural soil microbiome and a disc of leaf tissue from a plant inoculated with a microbiome from a fallow field (1, 3, or 16 years post-agriculture) in an arena. All choice tests were performed within plant species. Choices represent the microbiome treatment of the disc each larva was first observed to eat. **(B)** Probabilities (with 95% confidence intervals) of selecting the agriculture microbiome-inoculated plant averaged across plant species; the dashed line indicates an equal preference for agriculture- and fallow-microbiome treated plants. **(C)** Choices by crop species; light grey bars indicate the proportion of larvae that chose the plant treated with the agricultural soil microbiome whereas the dark grey bars indicate instances in which the fallow microbiome plant was chosen. N = 10-20 tests per plant species × fallow inoculant age level after omitting larvae that did not feed within the 2 h trial. An asterisk (*) indicates a significant preference.

Overall, the *S. frugiperda* larvae preferred to feed on plants inoculated with 16-year fallow versus agricultural soil microbiomes, but did not exhibit a significant preference for plants inoculated with the other fallow microbiomes (**Figure 5B**). Considering the species individually, the significant preference for late succession-treated plants was only observed for cucumbers, with the larvae choosing the agricultural microbiome plants with a probability of only 0.263 (95% CI: 0.1140 to 0.498) versus 16-year microbiome cucumber leaves (**Figure 5C**).

## Discussion

Overall, we found that soil microbiomes from varying stages of fallow succession can differentially affect both the pest resistance and growth of different crop species, but that the effects are species-specific, and often contrasting. In line with the pattern we previously observed in the *S. altissima* study that motivated this experiment (Howard et al., 2020), later succession microbiomes conferred greater *T. ni* resistance to the crop species, particularly cucumber (**Figure 2**). In contrast, tomato plants were least resistant to *S. frugiperda* when inoculated with late succession (16 year fallow) microbiomes (**Figure 4**). These species-specific responses to the various microbiomes were further illustrated through differences in plant growth. For example, lettuce produced the largest leaves when inoculated with a 3-year fallow microbiome, the microbiome in which cucumber performed worst, while tomato growth was overall unaffected by inoculant successional age. Collectively, these results indicate that various plant species have different phenotypic responses to different microbiomes and point to the need to study microbiome-influenced phenotypes in a broad and taxon-replicated range of plant species, as well as the underlying mechanisms of how microbiomes assemble and affect these plant traits.

These differential phenotypic responses of plant species to soil microbial communities of varying successional age could be due to differences in microbiome assembly, as well as the degree to which different plants rely on microorganisms for different functions. It is well known that different species, and even different cultivars and genotypes, of plants assemble distinct microbiomes (Lundberg et al., 2013; Peiffer et al., 2013; Cardinale et al., 2015; Fitzpatrick et al., 2018; Matthews et al., 2019). While few studies have directly compared microbiome assembly on different crop species, Matthews et al. (2019) recently found that maize, tomato, pea, and onion plants not only assembled microbiomes with distinct community structures, but also differed in the variability of their rhizosphere microbiome composition when grown in grassland versus woodland soils. In their study, tomato and maize showed the greatest variation in rhizosphere assembly at the individual taxon (OTU) level by soil type. Yet, even with differences in nutrients between the soils, they found that tomato and maize growth were unaffected, indicating that the performance of these species is robust and might not easily be altered by shifts in microbiomes. However, as most of the differentially abundant OTUs between the two soil types in their study came from the same taxonomic families and were potentially functionally redundant, it is difficult to assess whether these plant species differed from the other crops in their capacity to discriminate or actively influence their microbiome assembly (Matthews et al., 2019). On the other hand, we saw that maize, cucumber, and lettuce growth was affected by microbial community treatments, suggesting that these species might assemble even more divergent microbial communities under the different inoculation treatments and perhaps are not as able to actively select a microbiome. Similar to Matthews et al. (2019), we found that tomato growth was relatively robust to our microbiome treatments. This may have to do with its physiology, as tomato growth and reproduction are particularly resilient, even when defoliated (Slack, 1986). Thus, some species may be less likely than others to be affected by changes in their microbiomes due to their physiology. Plants also vary in their reliance on microbes for different functions related to growth and defense, some patterns of which may be phylogenetically generalizable (*e.g.*, Brassicaceae not forming symbioses with arbuscular mycorrhizal fungi for resource acquisition (Tester et al., 1987), many grasses hosting alkaloid-producing endophytes for herbivore defense (Clay, 1988). Thus, studying a diverse range of plant species, especially in a taxon-replicated manner, may help us understand and better predict plant responses to microbiomes.

While the differences between the patterns of herbivore resistance we saw here and in our previous study of *S. altissima* may be driven by the phylogenetic diversity of the plant hosts, some differences might also be due to comparing domesticated crops to wild plants. While *S. altissima* may be ecologically similar to a crop in that it grows in agriculturally-altered habitats, including fallow fields, its abilities to form microbial symbioses may not have been under the same selective pressures as plants that have been bred for agricultural performance. Comparisons between crops and their wild relatives have indicated that domestication has altered their interactions with microorganisms (Pérez-Jaramillo et al., 2018), sometimes resulting in plants that form symbioses with resource mutualists, such as arbuscular mycorrhizal fungi, less readily (Martín-Robles et al., 2018). Breeding under high-input agricultural regimes (*e.g.*, ample fertilizer) may make roots less conducive to forming microbial symbioses and relax selection on the ability to form symbioses—or even select against forming symbioses due to the costs of maintaining them when resources are abundant (Pérez-Jaramillo et al., 2016; Porter and Sachs, 2020). Furthermore, breeding crops for improved pathogen resistance may also inadvertently reduce the ability of plants to form symbioses with mutualistic microbes due to common pathways of colonization (Porter and Sachs, 2020). Wild sunflower accessions are slightly more readily colonized by mycorrhizae (Turrini et al., 2016) and also less resistant to pathogens than domesticated lines (Leff et al., 2016). With regard to the crops studied here, previous studies have indicated that cultivated lettuce and maize plants assemble different microbiomes than their wild relatives (Cardinale et al., 2015; Szoboszlay et al., 2015), and that cultivated tomato plants respond differently to soil conditioning compared to their wild counterparts (Carrillo et al., 2019). It is also important to note that these studies found substantial cultivar-level variation in the microbiomes of these crop species, which emphasizes the need to not only characterize the responses of different plant species to microbiomes, but also a diverse range of genotypes within species. In our study, we only examined one cultivar per crop species to maximize the number of species tested. This, however, limits our ability to make generalizations for specific species and assess the intraspecific variation among cultivars. For a general application of microbiome-enhanced plant resistance, studies examining crop responses to microbiomes should aim to capture inter-cultivar variation.

While we observed effects of soil microbiomes on plant resistance to insect pests, we did not investigate the basis of this herbivore resistance, limiting our ability to understand how these microbiomes are affecting the plants. For instance, the differences in weight gained by *S. frugiperda* feeding on plants inoculated with different microbiomes appears to be due to greater biomass accumulation efficiency rather than amount of tissue eaten, but we cannot tell if this is driven by differences in defense compounds (*e.g.*, digestibility reducers) or the nutritional value of the leaves. Interestingly, while tomato’s resistance to *S. frugiperda* was affected by the different microbiomes, its resistance to the other generalist noctuid pest we tested, *T. ni*, was not (**Figure 4**). Similarly, the microbiome treatments only affected cucumber’s *T. ni* resistance (**Figure 2**), indicating that not only are these microbiome-mediated resistance phenotypes plant species-specific, but also herbivore-specific, even as generalist (and here also confamilial) herbivores are typically expected to be similarly affected by chemical defenses (Ali and Agrawal, 2012). The data presented here suggest that different plant defense traits or trait combinations expressed by the same plant can contribute to the resistance to different herbivore species, even when the herbivore species are closely related. Consequently, the findings expand the interaction diversity hypothesis (Berenbaum and Zangerl, 1996; Iason et al., 2011) to include defense-related plant secondary metabolism that is mediated by the microbial community. This further suggests microbe-mediated changes in plant secondary metabolism as one of the drivers of functional chemical diversity in plants (Kessler and Kalske, 2018). Investigating the leaf chemistry of these microbiome-treated plants may help us understand whether these patterns of resistance are driven by different mechanisms. Additionally, we observed some discrepancies between our two measures of herbivore resistance that are difficult to resolve without understanding the underlying mechanisms of resistance. For example, plants treated with 3-year fallow microbiomes were overall more resistant than agricultural-microbiome plants to *T. ni* based on the no-choice assays (**Figure 2**), but the larvae exhibited a preference (albeit marginally significant) for them over the supposedly less resistant agricultural-microbiome plants in the choice experiments (**Figure 3B**). This discrepancy could be an artifact of using excised leaf discs in our assay, wherein defenses induced *via* the mechanical damage from cutting leaf tissue could result in the more defended leaves being more apparent, and thus more attractive to larval herbivores, than the less conspicuous, but actually less chemically-defended plants (Carroll et al., 2006).

Moreover, we do not know how, mechanistically, the microbiomes are contributing to the plants’ resistance phenotype. In addition to directly altering plants’ secondary metabolism, it is possible that pathogenic microbes in the soil (or non-pathogenic microbes perceived by the plants as pathogens) could be altering insect resistance by inducing salicylic acid-mediated responses and simultaneously suppressing the oft-reciprocally antagonistic jasmonic acid pathway that mediates defenses to chewing herbivores (Thaler et al., 2012). One recent study demonstrated that a rhizosphere-dwelling strain of *Pseudomonas* sp. that induced systemic pathogen susceptibility increased tomato plants’ resistance to *T. ni*, further indicating that the salicylic acid-jasmonic acid trade-off may play an important role in mediating rhizosphere microbial influence on insect herbivory (Haney et al., 2018). Another recent study (Blundell et al., 2020) implicated salicylic acid as an important mediator of soil microbe-influenced resistance to a hemipteran herbivore (which generally tend to be more strongly affected by salicylic acid- versus jasmonic acid-mediated defenses), further emphasizing the potential importance of rhizosphere microbes affecting plant-insect interactions through altering phytohormonal signaling. Thus, changes in the functional composition of soil microbial communities, including the abundance of pathogens—which are expected to shift over fallow succession (Hannula et al., 2017)—could affect herbivore resistance through altering plant defense responses. It is important to note, however, that since we performed our herbivore resistance assays with excised leaf tissue, we are likely masking potential differences in herbivore-induced resistance responses between our microbial treatments. While our study shows that soil microbiomes can differentially affect plants’ resistance to herbivores, the underlying mechanisms for these phenotypic shifts warrant further study.

With agricultural losses to pest damage expected to increase under a warming climate (Deutsch et al., 2018), investigating novel tools such as soil microbiome manipulations to improve the herbivore resistance of crop plants is becoming increasingly relevant (Pineda et al., 2017; Bell et al., 2019). In this study, we found that fallow agricultural fields may harbor soil microbiomes that can promote the growth and pest resistance of some crop plants. We found that, in comparison to agricultural microbiomes, the late succession soil microbiomes most notably improved cucumber’s resistance to *T. ni*, a pest which is especially damaging to cucumbers in greenhouses, where microbial inoculants would be relatively easy to apply (Sarfraz et al., 2011). This finding is in line with our previous work, which has suggested that successional shifts in soil microbiomes are an important factor driving increases in the herbivore resistance of a wild plant (Howard et al., 2020), yet the underlying mechanisms for this microbe-mediated resistance are still unresolved. However, it is becoming clearer that various early successional forbs and grasses (Pineda et al., 2020), as well as organic management practices (Blundell et al., 2020), can condition soil to promote herbivore resistance. Thus, in addition to providing related ecosystem services as habitat for natural enemies of pests (Denys and Tscharntke, 2002), fallow land may be worth investigating as sources of beneficial soil microbiomes that are adapted to local edaphic conditions, not only potentially improving their establishment and efficacy (Hawkes and Connor, 2017), but also reducing the non-target risks of introducing non-native microbes (Hart et al., 2018). Yet, sources aside, our study indicates that observing beneficial effects of an inoculant on one plant species may not be predictive of its capacity to improve the performance of another.

## Data Availability Statement

The data that support this paper are available in the Cornell eCommons repository (https://ecommons.cornell.edu/) under the title of this publication.

## Author Contributions

MH, JK-K and AK designed the study. MH and CM conducted the experiments and analyzed the data. MH drafted the manuscript with input from AK and JK-K.

## Funding

This research was supported by a Sustainable Biodiversity Fund grant from the Atkinson Center for a Sustainable Future, a Schmittau-Novak small grant from the School of Integrative Plant Science at Cornell University, a Toward Sustainability Foundation grant, an Undergraduate Minority Research grant from the College of Agriculture and Life Sciences at Cornell University, and a grant from NIFA Multistate NE-1501. MH was supported by a Horton-Hallowell Fellowship from Wellesley College and a Sellew Family Fellowship from Cornell University.

## Conflict of Interest

The authors declare that the research was conducted in the absence of any commercial or financial relationships that could be construed as a potential conflict of interest.
